# RETRACTION: Identification of IGF2 Promotes Skin Wound Healing by Co‐Expression Analysis

**DOI:** 10.1111/iwj.70659

**Published:** 2025-04-23

## Abstract

**RETRACTION:** LiuX. , TengY. , LiH. , LuoD. , LiH. , ShenJ. , DuS. , ZhangY. , WangD. , and JingJ. , “Identification of IGF2 Promotes Skin Wound Healing by Co‐Expression Analysis,” International Wound Journal 21, no. 4 (2024): e14862, 10.1111/iwj.14862.38572823 PMC10993366

The above article, published online on 4 April 2024, in Wiley Online Library (http://onlinelibrary.wiley.com/), has been retracted by agreement between the journal Editor in Chief, Professor Keith Harding; and John Wiley & Sons Ltd. Following an investigation by the publisher, all parties have concluded that this article was accepted solely on the basis of a compromised peer review process. The editors have therefore decided to retract the article. The authors did not respond to our notice regarding the retraction.

**RETRACTION:** X. Liu , Y. Teng , H. Li , D. Luo , H. Li , J. Shen , S. Du , Y. Zhang , D. Wang , and J. Jing , “Identification of IGF2 Promotes Skin Wound Healing by Co‐Expression Analysis,” International Wound Journal 21, no. 4 (2024): e14862, 10.1111/iwj.14862.38572823 PMC10993366

The above article, published online on 4 April 2024, in Wiley Online Library (http://onlinelibrary.wiley.com/), has been retracted by agreement between the journal Editor in Chief, Professor Keith Harding; and John Wiley & Sons Ltd. Following an investigation by the publisher, all parties have concluded that this article was accepted solely on the basis of a compromised peer review process. The editors have therefore decided to retract the article. The authors did not respond to our notice regarding the retraction.

